# Effects of Low-Fat and High-Fat Meals, with and without Dietary Fiber, on Postprandial Endothelial Function, Triglyceridemia, and Glycemia in Adolescents

**DOI:** 10.3390/nu11112626

**Published:** 2019-11-02

**Authors:** Corrie M. Whisner, Siddhartha S. Angadi, Nathan Y. Weltman, Arthur Weltman, Jessica Rodriguez, James T. Patrie, Glenn A. Gaesser

**Affiliations:** 1 College of Health Solutions, Arizona State University, 550 N Third St., Phoenix, AZ 85004, USA; cwhisner@asu.edu (C.M.W.); sangadi@asu.edu (S.S.A.); 2 Department of Anesthesiology, Perioperative and Pain Management, Brigham and Women’s Hospital, Boston, MA 02115, USA; nweltman@bwh.harvard.edu; 3 Department of Kinesiology, University of Virginia, Charlottesville, VA 22903, USA; alw2v@virginia.edu; 4 Soriant Solutions, Roswell, GA 30075, USA; jrvmsv@gmail.com; 5 Department of Public Health, University of Virginia, Charlottesville, VA 22903, USA; jp4h@virginia.edu

**Keywords:** flow-mediated dilation, glycemia, insulin, insoluble fiber, vascular, cardiovascular disease, pediatric, youth

## Abstract

The consumption of fiber-rich foods may negate the deleterious effects of high-fat meals on postprandial triglyceridemia and endothelial function. Despite supportive data in adults, little is known about the effects of high-fat and high-fiber foods on cardiovascular health parameters in pediatric populations. In this crossover trial, male and female adolescents (*n* = 10; 14.1 + 2.6 years; range 10–17 years) consumed (1) low-fat, low-fiber, (2) low-fat, high-fiber, (3) high-fat, low-fiber, and (4) high-fat, high-fiber breakfast meals in randomized order, each following an overnight fast. Baseline and 4 h post-meal blood was obtained for determination of glucose, insulin and triglyceride concentrations. Endothelial function was assessed via brachial artery flow-mediated dilation (FMD). Postprandial FMD was not significantly changed after any meal. However, regression analyses revealed a significant inverse relationship between the change in 4 h triglyceride concentration and change in 4 h FMD for the high-fat, low-fiber meal (β = −0.087; 95% CI = −0.138 to −0.037; *p* = 0.001) that was no longer significant in the high-fat, high-fiber meal (β = −0.044; 95% CI = −0.117 to 0.029; *p* = 0.227). Interpretation of these analyses must be qualified by acknowledging that between-meal comparison revealed that the two regression lines were not statistically different (*p* = 0.226). Addition of high-fiber cereal to the high-fat meal also reduced 4 h postprandial triglyceride increases by ~50% (*p* = 0.056). A high-fiber breakfast cereal did not attenuate postprandial glucose and insulin responses after consumption of a low-fat meal. While further work is needed to confirm these results in larger cohorts, our findings indicate the potential importance of cereal fiber in blunting the inverse relationship between postprandial hypertriglyceridemia and FMD after consumption of a high-fat meal in adolescents.

## 1. Introduction

Endothelial dysfunction is an underlying issue associated with cardiovascular disease risk, that directly relates to the pathogenesis of atherosclerosis [1]. It is well documented that the postprandial period may be important in the development of coronary artery disease [2,3]. The transient increase in postprandial triglycerides and glucose may play a key role in this relationship, as non-fasting triglycerides [4] and glucose [5,6] have been reported to be significant predictors of coronary artery disease. Because individuals in western societies spend a considerable amount of their time in a postprandial state, it may be important to identify foods that could potentially be cardioprotective during this period [7]. Although research into the importance of the postprandial period to atherosclerosis has largely focused on adults, this may also be relevant to the pediatric population, because atherosclerosis has been documented to begin in children as young as 6 years of age [8,9].

A meta-analysis indicated that ingestion of a mixed meal typically impairs endothelial function in adults, assessed by brachial artery flow-mediated dilation [10]. Brachial artery flow-mediated dilation (FMD) is the most widely used non-invasive technique to assess endothelial function in humans [11], and a meta-analysis demonstrated that impairment of the brachial artery FMD is significantly associated with future cardiovascular morbidity and mortality [12]. Impairment of the endothelial function could be due in large part to transient increases in postprandial triglycerides [13,14,15,16,17,18,19] and glucose [20].

Although several studies have shown that high-fat meals contribute to the transient impairment of endothelial function [13,14,15,16,17,18,19], this finding is not universal [21,22]. In adults, impairment in endothelial function after a high-fat meal has been reported to be significantly correlated with an increase in postprandial plasma triglycerides [13,14,15,17,18]. Two studies on adolescents, however, indicated that decrements in FMD after ingestion of a high-fat meal were not significantly correlated with postprandial triglyceridemia [23,24]. The type and quantity of fat ingested may influence this relationship [25]. Also influential is the inclusion of fiber-rich foods, which improve endothelial function [26,27] and have been associated with a reduced risk of cardiovascular disease [28,29,30].

We previously reported that in men and women with the metabolic syndrome, a low-fat, high-fiber meal acutely improved endothelial function, assessed by FMD, whereas a high-fat, low-fiber meal impaired endothelial function [26]. The high cereal content of the high-fiber meal, primarily from wheat bran [31,32,33,34], may have contributed to the salutary effects of this meal. Wheat fiber has also been reported to attenuate postprandial triglyceridemia, in response to a high-fat test meal [35,36,37]. Because postprandial impairment of endothelial function has been reported to be correlated with a postprandial increase in plasma triglycerides [13,14,15,17,18], we hypothesized that addition of cereal fiber to a high-fat meal would attenuate postprandial endothelial dysfunction, and that the magnitude of FMD attenuation would correlate with the reduction in postprandial triglycerides.

Although mixed meals [10], high-fat meals [13,14,15,16,17,18,19], and oral glucose loading [20] have all been shown to impair FMD in adults, limited data are available in younger populations. High-fat meals reduced postprandial FMD in adolescents [23,24,38], but glucose loading has been reported to have no effect on endothelial function in children and adolescents [39,40]. The high-fat meals of adolescents were 60–70% fat, relatively low in protein (5–13%), with a total energy content of 1000–1100 kcal [23,24,38]. These high-fat meals are not representative of typical meals consumed by adolescents. Notably, these meals were essentially devoid of dietary fiber, which may be important for preserving postprandial endothelial function [26]. Thus, in adolescents, the effects of meals with a more typical macronutrient composition on FMD are unknown. We chose to examine the effects of mixed meals, varying in carbohydrate, fat, and fiber content, on endothelial function in adolescents, to fill this research gap. This may be clinically relevant because atherosclerosis has been reported to begin in this age group [8,9]. We hypothesized that (1) a low-fat, high-fiber meal [26] would enhance postprandial FMD, (2) a high-fat, low-fiber meal would impair postprandial FMD, and (3) a high-fat meal supplemented with wheat bran would mitigate the expected impairment of FMD induced by the high-fat, low-fiber meal, and that this would be associated with a smaller postprandial increase in plasma triglyceride concentration.

## 2. Materials and Methods 

### 2.1. Participant Recruitment

This study was approved by the University of Virginia Institutional Review Board and carried out in accordance with the Declaration of Helsinki. Healthy male and female adolescents were recruited via internet postings, flyers, and referrals from health care professionals. Potential subjects underwent a screening visit to determine eligibility. Eligible participants were enrolled after obtaining minor assent and parental written informed consent. Participants were required to be between 8 and 17 years of age. Exclusion criteria included smoking; known food allergies; pregnancy; known cardiovascular or metabolic disease; use of medications that affect vascular function or blood pressure, cholesterol, or glucose; use of nutritional supplements other than a multivitamin; or following a diet designed for weight loss, or attempted weight loss, within the previous 3 months. Females of childbearing potential provided a urine sample for a pregnancy test. To control for the potential effects of menstrual cycle on vascular reactivity and blood lipids, postmenarcheal girls were studied in the early follicular phase (days 2–6) of the menstrual cycle. All subjects also underwent body composition assessment via air-displacement plethysmography (BOD POD body composition system; Life Measurement Instruments, Concord, CA, USA).

Ten healthy adolescents (three females, seven males), with ages in the range 10–17 years, participated in this study. An outline of participant characteristics is provided in Table 1.

### 2.2. Study Design and Interventions

Enrolled adolescents consumed four meal challenges (2 × 2 design) in random order: 1. low-fat, low-fiber; 2. low-fat, high-fiber; 3. high-fat, low-fiber; 4. high-fat, high-fiber. Meal challenges were provided as breakfast meals, following an overnight fast. Participants were given 15–20 min to consume the meal. The total energy content, macronutrient composition, and fiber content of each meal were determined using ProNutra software (Viocare Technologies, Princeton, NJ). Glycemic index was determined using ESHA Food Processor SQL Software (ESHA Research, Salem, OR). Meal details are presented in Table 2.

- The low-fat, low-fiber meal consisted of 31.0 g of low-fiber, wheat-based cereal (General Mills Golden Grahams); 245.0 g of non-fat milk; 124.5 g of orange juice; 65.0 g of refined, enriched-grain bread; 15.0 g of margarine; and 14.0 g of jelly.
- The low-fat, high-fiber meal consisted of 50.0 g of high-fiber, wheat-based cereal (Kellogg’s All-Bran Original); 245.0 g of non-fat milk; 124.5 g of orange juice; 68.0 g of whole-grain wheat bread; 15.0 g of margarine; and 14.0 g of jelly.
- The high-fat, low-fiber meal consisted of 88.0 g of eggs; 28.0 g of cheddar cheese; 34.0 g of turkey sausage; 244.0 g of whole milk; 124.5 g of orange juice; and 10.0 g of margarine.
- The high-fat, high-fiber meal consisted of 50.0 g of high-fiber, wheat-based cereal (Kellogg’s All-Bran Original); 88.0 g of eggs; 28.0 g of cheddar cheese; 34.0 g of turkey sausage; 244.0 g whole milk; 124.5 g of orange juice; 10.0 g of margarine; and 2.0 g sucralose sweetener.

For each of the four meals, the ratio of saturated fat to monounsaturated fat to polyunsaturated fat was 1.0:1.0:0.5. All meals were prepared by the General Clinical Research Center (GCRC) Research Dietitian. Participants completed a 24 h dietary recall on the night preceding their first GCRC visit. They were asked to consume the same foods and beverages during the 24 h preceding each of the subsequent three GCRC visits.

### 2.3. Clinical and Biochemical Assessments

During each of the four GCRC visits, blood samples were taken from an indwelling venous catheter in the antecubital vein at baseline (pre-meal) and 1, 2, 3, and 4 h postprandially. Brachial artery flow-mediated dilation (FMD) was measured at baseline prior to each breakfast meal and again at 2 and 4 h postprandially. The FMD technique utilizes B-mode ultrasonography to evaluate endothelial function, following a reactive hyperemic stimulus. The reproducibility and repeatability of this technique has been well established [41,42]. Brachial artery assessments were obtained using 2D and Doppler ultrasound measurements (HDI 5000, Philips Ultrasound, Andover, MA, USA), with a linear-array transducer, at a transmit frequency of 12 MHz. The image depth was initially set at 4 cm, and gain settings were adjusted to provide an optimal view of the anterior and posterior walls of the artery. Once optimal settings were obtained, they were kept constant throughout the study for each participant. The imaging was performed in the long axis, approximately 4 cm proximal of the antecubital fold in the anterior/medial plane. All imaging was performed using the same investigator, who was highly skilled at the FMD technique and blinded to the treatment conditions.

Upon arrival for the meal challenge visits (0700 h to 0900 h) subjects rested in a supine position for 30 min in a quiet, dimly lit, temperature-controlled room (22 °C to 23 °C) before baseline measurements were taken to ensure hemodynamic stability. Following this, the non-dominant arm was immobilized for ultrasound imaging. Heart rate was continuously monitored by a three-lead electrocardiogram, and image acquisition was gated to the R-wave. Baseline images were captured, after a segment with a clear anterior and posterior intimal interface between the lumen and vessel wall was identified for continuous 2D gray-scale imaging. Following this, a rapid inflation/deflation blood pressure cuff was placed 2 cm distal of the antecubital fold and inflated to 50 mm Hg above systolic blood pressure for 5 min to produce forearm ischemia. After five minutes, the cuff was rapidly deflated (reactive hyperemia), and digital still images were captured every 5 s, from 30 s to 120 s post cuff release, to determine peak dilation. In addition, the three highest consecutive peak values were used to determine the average dilation.

Brachial artery images subsequently underwent offline analyses with edge-detection and wall tracking software (Brachial Analyzer, Medical Imaging Applications, Iowa City, IA, USA). The digital images were acquired and analyzed in accordance with the guidelines offered by the International Brachial Artery Reactivity Task Force [43]. The same blinded reader was used for all studies, and the lumen−intima interface was used to determine vessel diameter. FMD was calculated as (peak diameter−baseline diameter/baseline diameter) × 100. In our laboratory, the intraobserver variability for determination of FMD is 2.93% [44].

Hourly blood samples were collected for glucose, insulin, and triglyceride concentrations. Insulin was assessed using the manufacturer protocol for the IMMULITE 2000 (Siemens Corporation, New York, NY, USA). Glucose was measured using the YSI 2300 Stat Plus (YSI Life Sciences, Inc., Yellow Springs, OH, USA) and triglycerides were measured using the Abbott Architect 8000 (Abbott Laboratories, Abbott Park, IL, USA).

### 2.4. Statistical Analyses

#### 2.4.1. Postprandial Blood Analyses

##### Glucose and Insulin

Postprandial 1 h blood glucose and plasma insulin responses were analyzed on the natural logarithmic scale, by the linear mixed effect (LME) model analysis of variance (ANOVA). For both glucose and insulin analyses, the logarithmic 1 h postprandial change in the response represented the LME model-dependent variable, and the meal consumed represented the LME model-independent variable. Linear contrasts of the LME model ANOVA least-squares means were constructed, to formally test whether the 1 h change in glucose and insulin differed between meals. Within-meal hypothesis testing was conducted by testing the null hypothesis that the mean change in log_e_ (response) is equal to zero. A two-sided *p* < 0.05 decision rule served as the null hypothesis rejection criterion for this test. Formal hypothesis testing for between-meal comparisons of the 1 h changes in blood glucose and plasma insulin were conducted by testing the null hypothesis that the mean 1 h change in the log_e_(response) is the same irrespective of the meal consumed. This null hypothesis was rejected based on a two-sided *p* < 0.05 decision rule, and both comparison-wise and multiple-comparison Bonferroni-corrected *p*-values are reported.

Blood glucose and plasma insulin area under curve (AUC) was calculated by the trapezoidal rule for the entire 4 h postprandial period. The total AUC measurements were then rescaled to the natural logarithmic scale and analyzed via linear mixed effects analysis of variance (ANOVA). Formal tests for between-meal comparisons of blood glucose and plasma insulin AUC were conducted by testing the null hypothesis that the log_e_ (AUC) is the same irrespective of the meal consumed. This null hypothesis was rejected based on a two-sided *p* < 0.05 decision rule, and both comparison-wise and multiple-comparison Bonferroni-corrected *p*-values are reported.

##### Triglycerides

Data for the change in 4 h plasma triglyceride concentrations from baseline were analyzed on the original scale of measure, by repeated measures ANOVA. Formal tests for between-meal comparisons were conducted by testing the null hypothesis that the 4 h change in triglycerides is the same irrespective of the meal consumed. This null hypothesis was rejected based on a two-sided *p* < 0.05 decision rule, and both comparison-wise and multiple-comparison Bonferroni-corrected *p*-values are reported.

##### Flow-Mediated Dilation (FMD)

2 h and 4 h changes in FMD were analyzed via linear mixed-effects analysis of covariance (ANCOVA). Dietary fiber, fat, time, fiber × fat interaction, fiber × time interaction, fat × time interaction, and fiber × fat × time interaction were seven sources of response-variable variation that were examined via ANCOVA, and the response-variable variability attributable to baseline heterogeneity in underlying FMD was also separated out and considered in the ANCOVA as covariate, variable-induced, response-variable variability. Formal tests for between-meal comparisons of 2 h and 4 h FMD were conducted by testing the null hypothesis that mean FMD is the same irrespective of the meal consumed. Null hypothesis tests were rejected based on a two-sided *p* ≤ 0.05 criteria, and both unadjusted *p*-values and Bonferroni-corrected *p*-values are reported for all between-meal comparisons.

##### Regression Analysis

The relationship between the 4 h change in triglyceride concentration and 4 h change in FMD was assessed by way of repeated measures regression analysis.

## 3. Results

### 3.1. Glycemic Response Data

**Figure 1:**
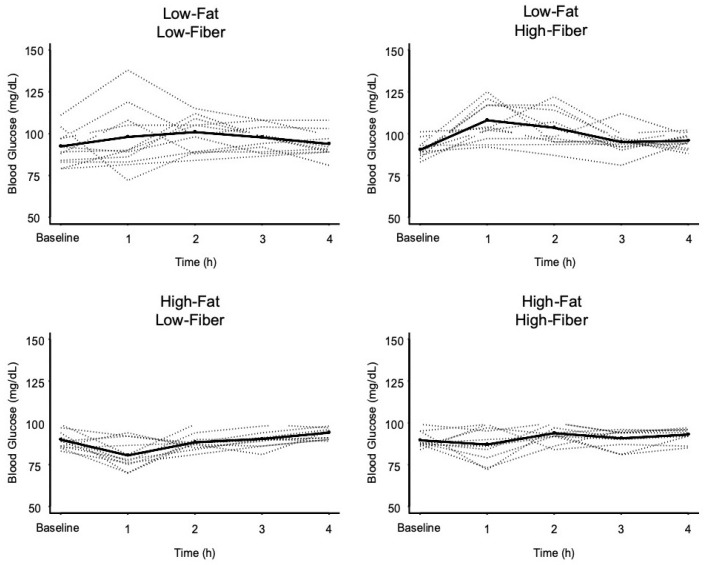
Blood glucose concentrations over time in response to the four meals. Dashed lines represent individual subject responses. Solid line represents mean response for all subjects. See text and Table 3 for statistical comparisons.

Individual and mean changes in blood glucose over time, in response to each meal, are presented in Figure 1, and results of statistical comparisons are presented in Table 3. Postprandial 1 h blood glucose was only elevated after the low-fat, high-fiber meal, from 91.1 ± 5.3 mg/dL to 108.4 ± 11.7 mg/dL. The 1 h blood glucose concentration after the high-fat, low-fiber meal was reduced by 12%, from 89.8 ± 5.4 mg/dL to 79.7 ± 9.6 mg/dL. The change in 1 h blood glucose was greater after the low-fat, high-fiber meal compared to both high-fat meals, but 1 h blood glucose was only lower for the high-fat, low-fiber meal compared to the low-fat, low-fiber meal.

**Figure 2:**
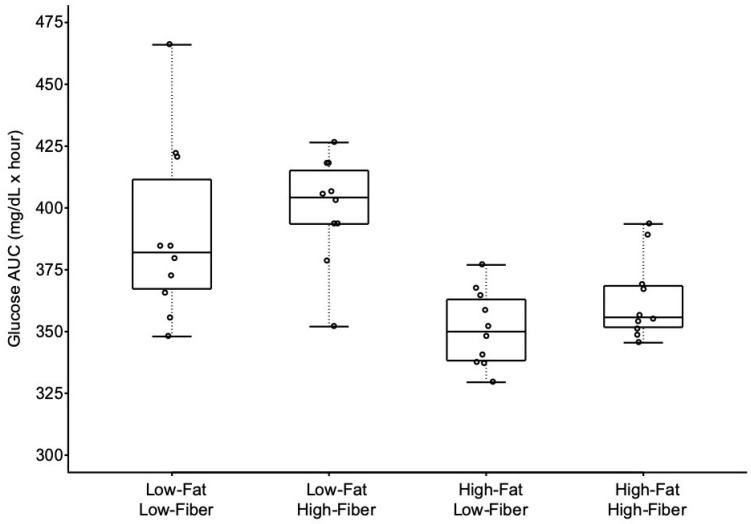
Glycemic response as area under curve (AUC) over 4 h in response to the four meals. Boxes for each meal represent the interquartile range. Minimum and maximum values are indicated at the tips of each vertical line. The median for each meal is depicted by the horizontal line within each box. See Table 4 for statistical comparisons between meals.

Blood glucose AUC differed between the four study meals (Figure 2 and Table 4). Mean blood glucose AUC was not different for the two low-fat meals, regardless of fiber content. Similarly, mean blood glucose AUC was not different for the two high-fat meals. Mean blood glucose AUC differed between high-fat, high-fiber and low-fat, low-fiber meals, but this difference disappeared after adjustment for multiple comparisons. The low-fat, high-fiber meal resulted in significantly greater glucose AUC, compared to both high-fat, low-fiber and high-fat, high-fiber meals.

### 3.2. Insulin Response Data

**Figure 3:**
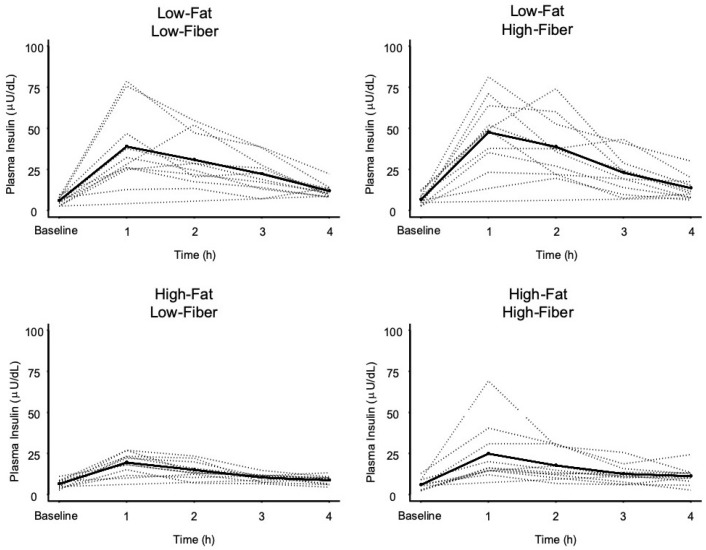
Plasma insulin concentrations over time in response to four meals. Dashed lines represent individual subject responses. Solid line represents mean response for all subjects. See text and Table 5 for statistical comparisons between meals.

Figure 3 shows the change in plasma insulin over time, in response to each meal, and results of statistical comparisons are presented in Table 5. All meals produced significant increases in plasma insulin. The 1 h increase in plasma insulin for the high-fat, low-fiber meal was significantly lower than the increase after both low-fat meals, regardless of fiber content. The two low-fat meals did not differ in 1 h postprandial insulin, despite a 6.4-fold greater fiber content in the low-fat, high-fiber meal.

**Figure 4:**
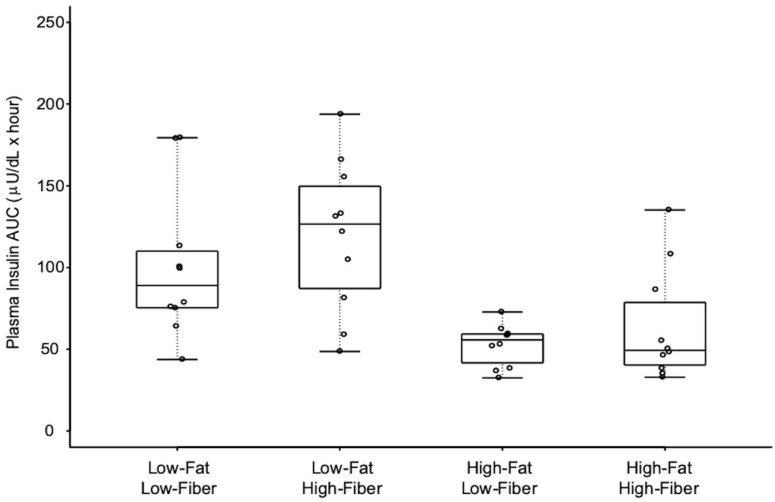
Plasma insulin as area under curve (AUC) over 4 h, in response to the four meals. Boxes for each meal represent the interquartile range. Minimum and maximum values are indicated at the tips of each vertical line. The median for each meal is depicted by the horizontal line within each box. See Table 6 for statistical comparisons between meals.

The mean 4 h plasma insulin AUC differed between the four meals (Figure 4 and Table 6). Mean plasma insulin AUCs were not different when comparing the two low-fat meals, but the low-fat, high-fiber meal resulted in significantly greater plasma insulin AUC compared to the high-fat, low-fiber meal and the high-fat, high-fiber meal. Mean 4 h plasma insulin AUC differed marginally between high-fat, high-fiber and low-fat, low-fiber meals, but this difference was not retained after adjustment for multiple comparisons.

### 3.3. Triglyceride Response Data

**Figure 5:**
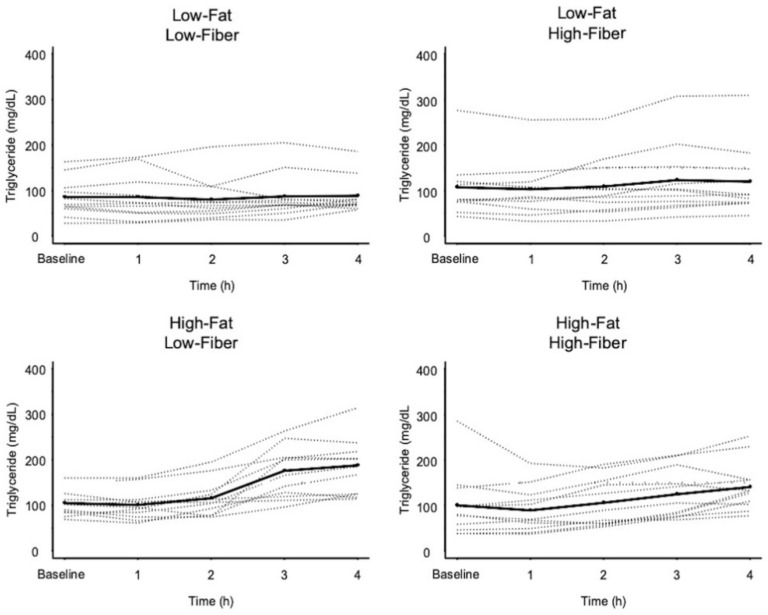
Plasma triglyceride responses to the four meals. Dashed lines represent individual subject responses. Solid line represents mean response for all subjects.

**Figure 6:**
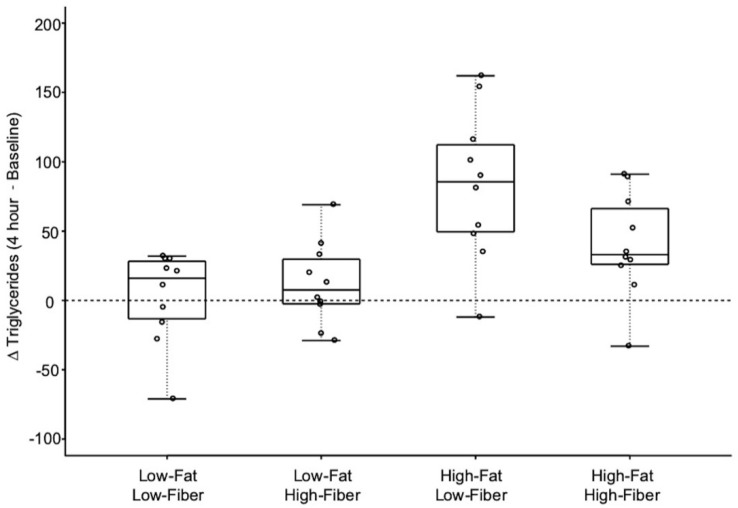
Change in plasma triglycerides over 4 h in response to the four meals. Minimum and maximum values are indicated at the tips of each vertical line. The median for each meal is depicted by the horizontal line within each box. See Table 7 for statistical comparisons.

Figure 5 shows the change in plasma triglycerides during the 4 h postprandial period for each meal. The mean for the 4 h change in triglyceride concentration differed significantly between the four meals (Figure 6 and Table 7). The mean 4 h change in triglyceride concentration was not different when comparing the two low-fat meals. The high-fat, low-fiber meal produced a greater increase in 4 h triglyceride concentration, compared to the low-fat, low-fiber meal and the low-fat, high-fiber meal. The addition of fiber to the high-fat, low-fiber meal marginally reduced the 4 h postprandial increase in triglycerides (*p* = 0.056).

### 3.4. Flow-Mediated Dilation

**Figure 7:**
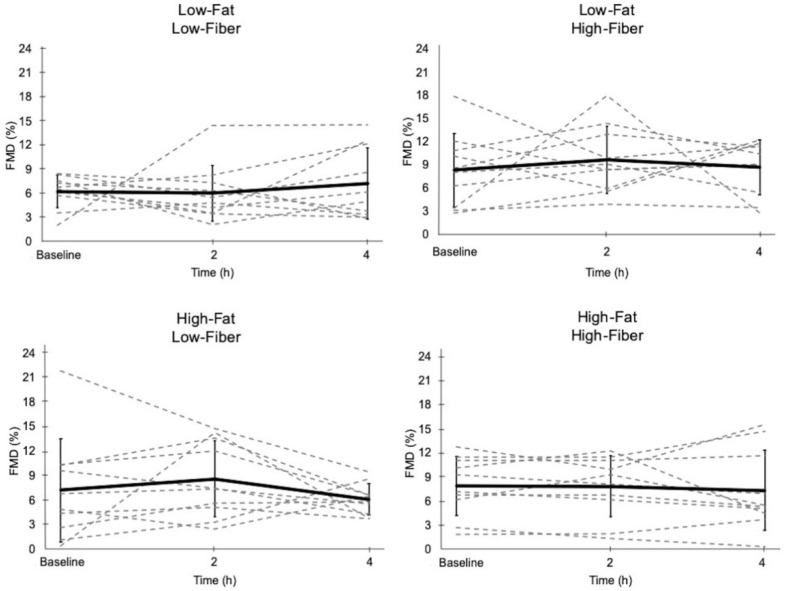
Endothelial function, as measured by flow-mediated dilation (FMD), following the four meals. Dashed lines represent responses for each individual. Solid line represents the mean response (±SD). FMD was not different across time points for all meals.

**Figure 8:**
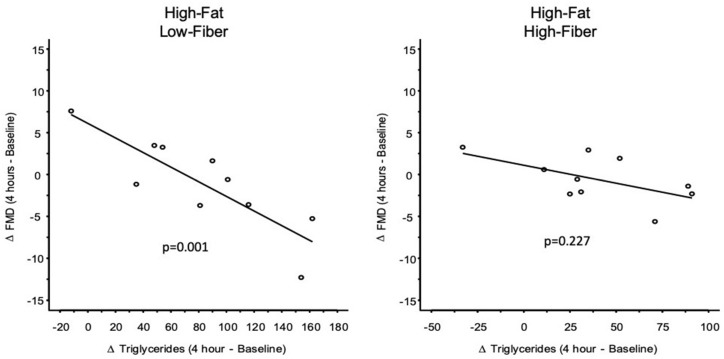
Attenuating effects of including a high-fiber cereal with a high-fat, low-fiber breakfast meal on the association between the change in 4 h plasma triglyceride concentration and change in 4 h FMD. For high-fat, low-fiber: β = −0.087 (95% CI = −0.138 to −0.037; *p* = 0.001). For high-fat, high-fiber: β = −0.044 (95% CI = −0.117 to 0.029; *p* = 0.227). Direct comparison of the two regression lines indicated that the slopes were not different from one another (*p* = 0.226).

Baseline and peak arterial diameters at each time point for each meal are presented in Table 8. Postprandial FMD was not affected by meals (Figure 7 and Table 8). Regression analysis revealed a significant inverse relationship between the change in 4 h triglyceride concentration and change in 4 h FMD only for the high-fat, low-fiber meal (Table 9 and Figure 8). Addition of a high-fiber cereal to the high-fat, low-fiber meal attenuated the slope of the regression line so that it was no longer statistically significant (Table 9 and Figure 8). Statistical comparison of the regression lines for the two high-fat meals in Figure 8 indicated that the slopes were not significantly different from one another (*p* = 0.226).

## 4. Discussion

There are two principal findings of this study. First, mixed-macronutrient breakfast meals of ~550–680 kcal did not elicit endothelial dysfunction in adolescents. Second, the addition of dietary fiber, primarily as wheat bran, to a high-fat meal attenuated the inverse relationship between 4 h postprandial increases in plasma triglyceride concentration, and changes in FMD during the same postprandial period. This was likely attributable in large part to the attenuated 4 h postprandial triglyceride response during the high-fat, high-fiber meal. From a risk mitigation perspective, insoluble fiber could be used adjunct with high-fat meals to blunt triglyceridemic excursions that are associated with post-prandial vascular dysfunction. This second conclusion requires some qualification, as discussed below.

Dietary fiber, however, failed to attenuate the postprandial rise in glucose and insulin concentrations following the ingestion of a low-fat meal. The rise in blood glucose following the two low-fat meals was relatively low, which may explain the lack of association between fiber content and postprandial glycemia. In adults, post-meal glucose concentrations may need to exceed ~122 mg/dL to impair FMD [45].

A meta-analysis by Thom et al. [10] indicated that FMD was generally impaired after meal ingestion, but details of the macronutrient composition of meals were not well described in the included studies. Further, data on adolescents are limited and restricted to feeding trials that are not typical (i.e., oral glucose tolerance tests, and very high-fat meals that are low in protein and carbohydrates). To address these gaps, we used carefully designed meals among adolescents to study the effects of macronutrient composition on postprandial FMD. We hypothesized that (1) a low-fat, high-fiber meal [26] would enhance postprandial FMD; that (2) a high-fat, low-fiber meal would impair postprandial FMD and that; (3) a high-fat meal, supplemented with wheat bran, would mitigate the expected impairment of FMD induced by the high-fat, low-fiber meal, and that this would be associated with an attenuated postprandial increase in plasma triglycerides. Only the third hypothesis was accepted, as evidenced by data showing that the addition of a high-fiber cereal to a high-fat meal attenuated the significant inverse relationship between the postprandial increase in triglycerides and decrease in FMD, such that it was no longer significant. Regression analysis demonstrated that the slope of the decrement in FMD with increases in postprandial plasma triglyceride concentration was reduced by 50%. However, acceptance of our third hypothesis requires qualification. Despite the within-meal results depicted in Figure 8, direct comparison of the slopes of the two regression lines indicated that they were not statistically different from one another. Based on our data, a post-hoc power analysis revealed that if a new study was designed to compare these two regression slopes, a sample size of 64 subjects would be required in order to have 0.80 power to detect the observed difference in slope parameters.

An inverse relationship between the postprandial changes in triglycerides and FMD has been reported previously in adults [13,14,15,17,18], but not in adolescents [23,24]. To the best of our knowledge, the current study is the first to show this inverse relationship between the postprandial changes in plasma triglycerides and FMD in adolescents. Our results, and the reports on adults, suggest that reducing the postprandial increase in triglycerides could reduce the deleterious impact of hypertriglyceridemia on endothelial function. Cereal fiber has been shown to attenuate postprandial triglyceridemia in adults [35,37]. Our data are the first to show that this may occur in a pediatric population (albeit a marginally significant unadjusted reduction, see Table 7). This may have salutary effects on reducing the potential impairment of endothelial function associated with a high-fat, low-fiber meal.

The magnitude of the postprandial increase in triglycerides is in part dependent upon the amount of fat ingested [37]. The increase in triglycerides after ingestion of the high-fat, low-fiber meal (105.2 ± 30.9 mg/dL to 188.1 ± 61.9 mg/dL) in this study is consistent with what would be expected on the basis of the amount of fat (~35 g) in our high-fat, low-fiber meal [37]. The addition of cereal fiber to our high-fat meal reduced the 4 h increase in triglycerides by ~50%. To date, the majority of data showing an inverse relationship between increases in triglycerides and decreases in FMD have been regarding adults. In healthy middle-aged men and women who consumed a high-fat meal (803 kcal; 53.4 g fat), Bae et al. [13] reported that postprandial impairment of FMD was significantly correlated (r = −0.65) with the increase in postprandial triglycerides. The inverse relationship between increased triglycerides and impairment in endothelial function after a high-fat meal has also been reported by others [13,14,15,17,18]. Bae et al. [13] also concluded that postprandial hypertriglyceridemia caused endothelial dysfunction via oxidant stress, as evidenced by a positive correlation between postprandial triglycerides and superoxide anion formation. Cereal fiber has potent antioxidant characteristics [46]. Our study is consistent with this hypothesis and suggests that the reduction in FMD associated with exaggerated postprandial triglyceridemia may be due to the antioxidant properties of the cereal fiber added to the high-fat, low-fiber meal.

Contrary to our hypothesis, the high-fat, low-fiber meal did not produce an impairment in FMD. This may be due to a number of factors. First, our test meal included 35 g protein, and it has been reported that endothelial dysfunction induced by a high-fat meal may be neutralized by the addition of protein to the meal [47]. Previous studies among adolescents provided much higher energy (1000–1100 kcal) meals, that contained 60%–70% of kcals from fat, with only 5–13% of kcals from protein [23,24,38]. This is more than twice the amount of fat served in our breakfast meal. These meals may not have been representative of typical meals consumed by adolescents. Thus, our data provide important insights into the effects of meals with a more typical macronutrient composition on FMD. Although there may be no universally acceptable definition of a “typical” meal for adolescents, our meals consisted of commonly consumed foods (e.g., cereal, milk, orange juice, bread, eggs, cheese, sausage). Also, based on data from adults [10], postprandial FMD impairment is less likely in people with baseline FMD < 10%, and when fasting glucose is normal. Our study cohort was generally healthy, which may have made it difficult to evaluate the influence of macronutrient composition on FMD. Future studies may benefit from studying the effects of fiber among adolescents with an elevated risk of obesity and cardiometabolic disease.

Repetitive postprandial hypertriglyceridemia may contribute to the development of coronary artery disease [13]. Reduction in cardiovascular disease risk has been associated with fiber intake [30]. This positive outcome of dietary fiber consumption may, in part, be related to the attenuation of triglycerides in the postprandial state, a period important in the development of coronary artery disease [2,3]. Atherosclerosis may begin as early as childhood and adolescence [8,9]. Therefore, as individuals in western societies spend a large amount of time in a postprandial state [7], it is imperative that cardioprotective dietary approaches are followed during childhood, when continued growth and development require increased energy consumption and more frequent eating episodes.

A secondary aim of this study was to evaluate whether the addition of fiber to both low-fat and high-fat breakfast meals would attenuate postprandial glucose or insulin responses. Although our data did not indicate a benefit for postprandial glycemia, data from adults have shown that the addition of dietary fiber from cereal grains reduces the risk of diabetes [48,49]. Studies among both normoglycemic overweight/obese and diabetic (Type 2) adults suggest that the consumption of dietary fiber, i.e., from oats and psyllium, improves postprandial insulin and glucose concentrations [50,51,52]. Despite consistent findings among adults, data on the effects of dietary fiber consumption in younger populations at risk of diabetes or reduction in glycemia and/or the insulin response are quite limited [53]. Available data for this understudied, yet important, period of life reveal mixed findings. Among children and adolescents with type 2 diabetes, psyllium fiber was effective at decreasing postprandial glycemia [54], while another study in adolescents found no effect of fiber intake on glucose metabolism [55]. Overall, conflicting findings and the gross lack of data among pediatric cohorts suggest a need for further investigation into the effects of fiber on the postprandial glycemic responses in adolescents.

### Strengths and Limitations

Given the paucity of data on the effects of dietary fiber on postprandial metabolic and vascular responses in younger populations, our focus on this population is a significant strength of the study. While many studies evaluate the effects of a single food or nutrient, this study evaluated the effects of fiber in the context of standard breakfast meals with realistic macronutrient composition. This increases the generalizability of findings to real-world settings, where foods or single nutrients are infrequently consumed in isolation. All four meals in this study were designed to provide similar macronutrient and total energy composition, so that the effect of fiber could be carefully studied. Although this is a strength, as it reduces variability associated with free-living conditions, we acknowledge that this approach may not reflect real-world conditions. Other strengths of this study include a protocol that involved carrying out strictly controlled and well thought out laboratory protocols. For example, FMD was measured up to 4 h after meal consumption. By extending this measure beyond the more typically observed 1–3 h in other studies [10], these data provide better insight into the effects of meal consumption on endothelial function. Further, a recent meta-analysis concluded that the majority of studies evaluating dietary effects on endothelial function reported limited or no details regarding the macronutrient, fiber and energy content of meals [10], thereby adding further value to the current study.

This study has some limitations. Primarily, the sample size was relatively small, which did not allow for sex comparisons, and also resulted in some of the statistical comparisons lacking significance after adjustment for multiple comparisons. For FMD, our primary outcome, we performed post-hoc sample size calculations based on the mean and SD data from the current study (Table 8). To reject the null hypothesis that Δ4 h FMD = 0, the required sample sizes for each meal exceeded 160 subjects. Even if statistically significant, the clinical relevance of such small changes in postprandial FMD (Table 8) is questionable. This reaffirms one of our principal findings, that mixed-macronutrient meals, such as those used in the current study, are not likely to induce endothelial dysfunction in adolescents. The meals were the same for each participant regardless of body weight, which could have influenced the results. However, for the high-fat, low-fiber meal, which resulted in the greatest increase in plasma triglycerides, the postprandial increase in triglycerides at 4 h was not significantly correlated with body weight (*r* = −0.24; *r*^2^ = 0.06). Our FMD data were not adjusted for shear rate. Although the normalization of FMD for shear has been recommended [11], the issue of normalizing FMD to shear is unresolved [11,56], and is age-dependent [57]. In children, the magnitude of brachial artery FMD has been reported to be unrelated to four different indices of shear rate [57]. In adolescents, Bond et al. [38] reported that postprandial changes in FMD were unrelated to shear rate AUC. Our study was carried out under strict laboratory conditions and only following acute meal challenges, which may limit the generalizability of data to real-world settings. This could have affected the interpretation of FMD results, as this measure undergoes circadian variation [44,58,59]. Lastly, although our data provide compelling evidence for the protective role of insoluble fiber, these data need to be replicated following chronic high-fat feeding.

## 5. Conclusions

Previous studies on children and adolescents have used either very high-fat meals or glucose loading to examine the effects of diet on FMD. This is the first study to look at the effects of low-fat meals (either low or high in fiber) on FMD in adolescents, and the first to include a high-fat meal to which dietary fiber was added. Thus, our study design is very novel, including four meals of varying macronutrient composition that have real-world applicability (i.e., neither extremely high in fat, nor relying solely on glucose loading to examine postprandial responses). We found that, overall, postprandial endothelial dysfunction was not evident in adolescents who ingested mixed-macronutrient meals consisting of commonly consumed foods. That the low-fat meals did not impair FMD is consistent with previous data showing a lack of FMD impairment following oral glucose tolerance tests in children and adolescents [39,40], and also with studies showing that low-fat meals do not impair FMD in adults [13,26]. More importantly, we found that the addition of high-fiber cereal to a high-fat breakfast meal lessened the postprandial triglyceride increase, such that the inverse relationship between postprandial triglyceridemia and FMD was no longer significant, as observed with the high-fat, low-fiber meal. This conclusion must be qualified by acknowledging that even though the within-meal regression results support this interpretation, statistical comparison of the two regression lines indicated that the slopes were not different. Nevertheless, these results are very novel, and provide information, for the first time, on the effects of meals with a range of macronutrient compositions on FMD and postprandial glucose, insulin, and triglycerides in adolescents. Further research is needed to understand how meal composition influences these parameters over a longer period of time, and if additional benefits are observed in adolescents with obesity and/or diabetes.

## Figures and Tables

**Table 1:** Participant anthropometric characteristics at study baseline (*n* = 10).

Subject Characteristic	Mean ± SD	Range
Age (years)	14.1 ± 2.6	10–17
Weight (kg)	48.6 ± 12.2	29.6–65.4
Height (cm)	163.3 ± 15.4	135.3–189.2
Body Mass Index Percentile	28.3 ± 16.5	9–50
% body fat	16.4 ± 7.2	8.1–28.8
Blood glucose (mg/dL)	90.6 ± 6.5	84.3–98.3
Plasma insulin (µU/mL)	6.2 ± 3.0	3.2–9.6
Plasma triglycerides (mg/dL)	99.6 ± 53.8	48.8–221.0

**Table 2:** Composition of study meals.

Nutritional Component	Low-Fat Low-Fiber	Low-Fat High-Fiber	High-Fat Low-Fiber	High-Fat High-Fiber
Energy (kcal)	545	546	550	680
Total Fiber (g)	3.0	19.3	0.3	16.3
Total Carbohydrate (g) ^a^	95.7 68.9%	103.7 64.1%	24.7 17.9%	61.7 27.7%
Total Fat (g)	11.4	12.1	34.6	36.3
Total Protein (g)	16.7	21.8	35.0	41.3
Glycemic Index	61	53	42	48

^a^ Percentages in the lower row represent the percent of energy for available carbohydrates in the meal after accounting for the lower, but non-negligible, energy yield from dietary fiber.

**Table 3:** Blood glucose changes from baseline to 1 h postprandial: comparison between meals.

Meal	Glucose mg/dL (Mean ± SD)		Low-Fat Low-Fiber	Low-Fat High-Fiber	High-Fat Low-Fiber	High-Fat High-Fiber
	Baseline	1 h	Unadjusted *p*-Values (Bonferroni-Adjusted *p*-Values)			
Low-Fat Low-Fiber	91.9 ± 10.5	98.4 ± 19.3	-	0.108 (0.647)	0.015 (0.090)	0.199 (1.000)
Low-Fat High-Fiber	91.1 ± 5.3	108.4 ± 11.7 *		-	<0.001 (<0.001)	0.002 (0.009)
High-Fat Low-Fiber	89.8 ± 5.4	79.7 ± 9.6 **			-	0.126 (0.756)
High-Fat High-Fiber	89.6 ± 4.9	87.0 ± 10.3				-

SD = Standard deviation; * Significantly higher than baseline blood glucose for that meal (*p* = 0.001); ** Significantly lower than baseline blood glucose for that meal (*p* = 0.009).

**Table 4:** Blood glucose area under curve (AUC): comparisons between meals.

Meal	Glucose AUC mg/dL × h (Mean ± SD)	Low-Fat Low-Fiber	Low-Fat High-Fiber	High-Fat Low-Fiber	High-Fat High-Fiber
		Unadjusted *p*-Values (Bonferroni-Adjusted *p*-Values)			
Low-Fat Low-Fiber	390 ± 36	-	0.454 (1.000)	0.006 (0.035)	0.045 (0.269)
Low-Fat High-Fiber	400 ± 22		-	<0.001 (<0.001)	0.001 (0.003)
High-Fat Low-Fiber	351 ± 16			-	0.110 (0.663)
High-Fat High-Fiber	363 ± 17				-

AUC = Area under curve for entire 4 h postprandial period; SD = Standard deviation.

**Table 5:** Plasma insulin responses 1 h postprandial: comparison between meals.

Meal	Insulin µU/mL (Mean ± SD)		Low-Fat Low-Fiber	Low-Fat High-Fiber	High-Fat Low-Fiber	High-Fat High-Fiber
	Baseline	1 h	Unadjusted *p*-Values (Bonferroni-Adjusted *p*-Values)			
Low-Fat Low-Fiber	5.9 ± 2.3	38.6 ± 22.3 *	-	0.682 (1.000)	0.048 (0.289)	0.169 (1.000)
Low-Fat High-Fiber	6.8 ± 3.4	45.7 ± 19.5 *		-	0.019 (0.112)	0.071 (0.426)
High-Fat Low-Fiber	6.1 ± 2.7	20.4 ± 6.4 *			-	0.407 (1.000)
High-Fat High-Fiber	5.8 ± 3.5	24.8 ± 18.0 *				-

SD = Standard deviation; * Significantly higher than baseline blood glucose for that meal (*p* < 0.001).

**Table 6:** Plasma insulin area under curve (AUC): comparisons between meals.

Meal	Insulin AUC µU/mL × h (Mean ± SD)	Low-Fat Low-Fiber	Low-Fat High-Fiber	High-Fat Low-Fiber	High-Fat High-Fiber
		Unadjusted *p*-Values (Bonferroni-Adjusted *p*-Values)			
Low-Fat Low-Fiber	97 ± 46	-	0.318 (1.000)	0.006 (0.035)	0.042 (0.251)
Low-Fat High-Fiber	119 ± 47		-	0.001 (0.003)	0.006 (0.035)
High-Fat Low-Fiber	55 ± 14			-	0.680 (1.000)
High-Fat High-Fiber	64 ± 35				-

AUC = Area under curve for entire 4 h postprandial period; SD = Standard deviation.

**Table 7:** Change in plasma triglyceride concentration from baseline to 4 h postprandial: comparison between meals.

Meal	Δ Triglycerides [4 h–Baseline] mg/dL (Mean ± SD)	Low-Fat Low-Fiber	Low-Fat High-Fiber	High-Fat Low-Fiber	High-Fat High-Fiber
		Unadjusted *p*-Values (Bonferroni-Adjusted *p*-Values)			
Low-Fat Low-Fiber	3 ± 33	-	0.515 (1.000)	0.001 (0.007)	0.030 (0.181)
Low-Fat High-Fiber	12 ± 30		-	0.003 (0.016)	0.083 (0.497)
High-Fat Low-Fiber	83 ± 54 *			-	0.056 (0.336)
High-Fat High-Fiber	40 ± 38 **				-

SD = Standard deviation; * Significant increase from baseline (*p* = 0.001). ** Significant increase from baseline (*p* = 0.008).

**Table 8:** Brachial artery diameters and flow-mediated dilation at each time point for the four meals.

		Baseline Diameter (mm)	Peak Diameter (mm)	FMD (%)
Low-Fat, Low-Fiber	0 h	3.00 ± 0.34	3.19 ± 0.39	6.2 ± 2.1
	2 h	3.10 ± 0.32	3.28 ± 0.37	6.0 ± 3.5
	4 h	3.11 ± 0.37	3.32 ± 0.36	7.2 ± 4.5
Low-Fat, High-Fiber	0 h	2.93 ± 0.43	3.16 ± 0.36	8.3 ± 4.7
	2 h	3.00 ± 0.32	3.28 ± 0.37	9.7 ± 4.4
	4 h	3.07 ± 0.45	3.32 ± 0.41	8.7 ± 3.6
High-Fat, Low-Fiber	0 h	2.91 ± 0.32	3.11 ± 0.30	7.2 ± 6.3
	2 h	3.03 ± 0.34	3.28 ± 0.29	8.6 ± 4.7
	4 h	3.14 ± 0.30	3.33 ± 0.35	6.1 ± 1.9
High-Fat, High-Fiber	0 h	2.91 ± 0.34	3.13 ± 0.31	7.9 ± 3.7
	2 h	3.05 ± 0.37	3.28 ± 0.36	7.8 ± 4.1
	4 h	3.06 ± 0.36	3.28 ± 0.34	7.3 ± 5.0

FMD: Flow-mediated dilation.

**Table 9:** Regression between changes in 4 h plasma triglycerides and 4 h FMD for the four meals.

Meal	Intercept [95% CI]	Slope [95% CI]	*p*-Value
Low-Fat, Low-Fiber	0.924 [−1.675, 3.524]	0.029 [−0.054, 0.111]	0.482
Low-Fat, High-Fiber	1.127 [−1.689, 3.943]	−0.060 [−0.152, 0.032]	0.191
High-Fat, Low-Fiber	6.144 [1.204, 11.083]	−0.087 [−0.138, −0.037]	0.001
High-Fat, High-Fiber	1.174 [−2.723, 5.071]	−0.044 [−0.117, 0.029]	0.227

FMD = Flow-mediated dilation; CI = Confidence interval.
